# TREM2 deficiency inhibits microglial activation and aggravates demyelinating injury in neuromyelitis optica spectrum disorder

**DOI:** 10.1186/s12974-023-02772-3

**Published:** 2023-04-03

**Authors:** Yun-Fan You, Man Chen, Yue Tang, Wen-Xiang Yu, Xiao-Wei Pang, Yun-Hui Chu, Hang Zhang, Ke Shang, Gang Deng, Luo-Qi Zhou, Sheng Yang, Wei Wang, Jun Xiao, Dai-Shi Tian, Chuan Qin

**Affiliations:** 1 Department of Neurology, Tongji Hospital, Tongji Medical College, Huazhong University of Science and Technology, Wuhan, 430030 People’s Republic of China; 2 Hubei Key Laboratory of Neural Injury and Functional Reconstruction, Huazhong University of Science and Technology, Wuhan, 430030 China

**Keywords:** TREM2, Microglia, Neuromyelitis optica spectrum disorder, Demyelination

## Abstract

Neuromyelitis optica spectrum disorder (NMOSD) is an inflammatory demyelinating disorder of the central nervous system (CNS) triggered by autoimmune mechanisms. Microglia are activated and play a pivotal role in response to tissue injury. Triggering receptor expressed on myeloid cells 2 (TREM2) is expressed by microglia and promotes microglial activation, survival and phagocytosis. Here, we identify a critical role for TREM2 in microglial activation and function during AQP4-IgG and complement-induced demyelination. TREM2-deficient mice had more severe tissue damage and neurological impairment, as well as fewer oligodendrocytes with suppressed proliferation and maturation. The number of microglia clustering in NMOSD lesions and their proliferation were reduced in TREM2-deficient mice. Moreover, morphology analysis and expression of classic markers showed compromised activation of microglia in TREM2-deficient mice, which was accompanied by suppressed phagocytosis and degradation of myelin debris by microglia. These results overall indicate that TREM2 is a key regulator of microglial activation and exert neuroprotective effects in NMOSD demyelination.

**Graphical Abstract:**

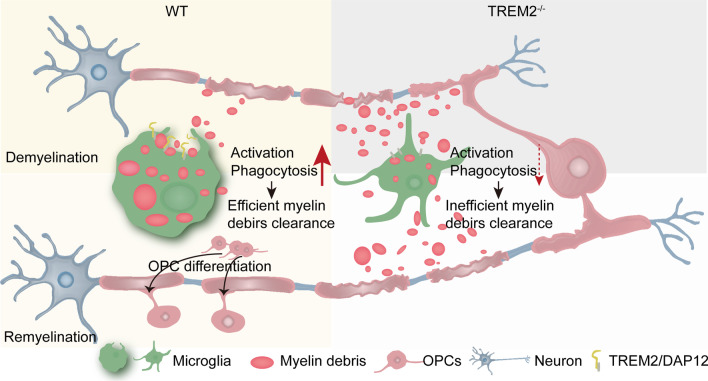

**Supplementary Information:**

The online version contains supplementary material available at 10.1186/s12974-023-02772-3.

## Background

Neuromyelitis optica spectrum disorder (NMOSD) is an inflammatory demyelinating disorder of the central nervous system (CNS). About 80% of patients are seropositive for immunoglobulin G (IgG) against an important water channel protein, aquaporin-4 (AQP4) on astrocytes [1]. This autoantibody then led to complement-dependent cytotoxicity and antibody-dependent cell-mediated cytotoxicity, resulting in oligodendrocyte damage, demyelination, and neurological deficits in NMOSD [2]. Demyelination generates a lot of myelin debris, which can selectively prevent oligodendrocyte precursor cells (OPCs) differentiation, which in turn aggravate demyelination, leading to myelin debris accumulation, and axonal damage [3]. Therefore, the effective removal of myelin debris is necessary for remyelination [4].

Microglia are innate immune cells that serve as the primary phagocytes in the CNS [5]. Studies have demonstrated that microglia play a significant role in the remyelination process in numerous CNS diseases including NMOSD. These microglial cells have been shown to participate in the phagocytosis of debris from damaged myelin, thereby clearing the affected tissue and promoting the repair process [6, 7]. This highlights the complex role that microglia play in NMOSD, where they can contribute to both the development of the disorder as well as its resolution.

Triggering receptor expressed on myeloid cells 2 (TREM2) is a protein expressed on the surface of microglia in CNS. It has a crucial role in regulating immune responses in the CNS and has been implicated in the pathogenesis of several neurodegenerative diseases, including Alzheimer's disease, Parkinson's disease, and multiple sclerosis [8]. Research has established TREM2 as a direct receptor for several signals generated by damaged myelin sheaths, including phosphatidylserine, sphingomyelin, and sulfatide. TREM2 acts as a sensor of the microenvironment in the CNS and is involved in the regulation of microglial activation, migration, and phagocytosis [9]. However, the specific role of TREM2 in microglial activation and dysfunction in NMOSD is limited.

Here, we explore the effect of TREM2 deficiency on microglia in the mouse model of NMOSD. In this model, demyelination was induced by stereotactic injection of AQP4-IgG and complement, which triggered microglial activation, proliferation, and removal of myelin debris. We further showed that TREM2 deficiency increased brain lesions in the mouse model of NMOSD. TREM2 defects reduced the clearance of myelin debris by microglia, leading to hindered OPCs differentiation and ultimately failure of myelin regeneration. Our findings demonstrate the important role of TREM2 in promoting myelin regeneration, suggesting that it is a potential therapeutic target for NMOSD.

## Materials and methods

### AQP4-IgG purification

Serum was collected from four patients diagnosed with AQP4-IgG seropositive NMOSD. Clinical data of NMOSD patients are provided in Additional file 1: Table S1. IgG was separated by protein G-agarose and prepared as a lyophilized powder as described [10, 11]. The lyophilized powder of IgG was dissolved in phosphate-buffered saline (PBS) at pH 7.4 and sterile filtered. The IgG concentration was adjusted to 20 mg/mL and named AQP4-IgG. This study was approved by the Ethics Committee of Tongji Hospital of Huazhong University of Science and Technology (TJ-IRB20190502).

### NMOSD animal model procedure

In all experiments, 8–12 weeks weight-matched (23–28 g) female C57BL/6 wild-type (WT) and TREM2^−/−^ mice (a generous gift from Professor Marco Colonna, Washington University, St. Louis, USA) were used. Animal experiments were approved by the Animal Care Committee of Tongji Hospital of Huazhong University of Science and Technology (TJH-202201008). Mice were maintained in air-filtered cages and fed normal mouse chow.

To induce NMOSD pathology as described previously [10–12]. Mice were anesthetized with isoflurane and mounted on a stereotaxic frame (RWD Life Science, Shenzhen, China). A midline scalp incision was made and a burr hole of diameter 1 mm was drilled in the skull 2-mm right of bregma. A 33-gauge needle attached to a 25-µl Hamilton syringe (Hamilton, Reno, NV, USA) was inserted 3 mm deep to infuse 6 μl of AQP4-IgG and 4 μl of human complement at a rate of 0.5 μl/min. After the injection, the needle remained in place for another 10 min. The syringe was then removed and the scalp was closed using a 4–0 nylon suture.

### Western blot

Neutral red-labeled lesions were dissected from the mouse brain as described previously [11, 13]. The tissue was lysed in RIPA buffer (Beyotime, China) supplemented with phosphatase inhibitors (Beyotime, China). Tissue lysates were centrifuged at 12000 *g* for 15 min at 4 °C. Supernatant was collected and protein concentration was determined. Total protein (20–40 μg) was added in 10% sodium dodecyl sulfate–polyacrylamide gels and blotted to 0.45 μm nitrocellulose (NC) filter membranes (Boster, China). The membranes were blocked with 5% skim milk for 1 h at room temperature and then incubated overnight with primary antibodies. The NC membranes were washed three times with Tris-buffered saline with 0.05% Tween-20 and incubated for 1 h at room temperature with horseradish peroxidase-labeled secondary antibody. A complete list of antibodies used is shown in Additional file 1: Table S2. Target proteins were visualized with enhanced chemiluminescence reagents and evaluated via a CCD camera (BLT, GelView 6000pro).

### Immunofluorescence microscopy and image analysis

Brains were collected for frozen sections at 7 days post-injection. Mice were anesthetized with isoflurane and transcardially perfused with 30 mL ice-cold PBS, followed by 20 mL 4% paraformaldehyde. Brains were post-fixed overnight in 4% paraformaldehyde and then gradient dehydration with 30% sucrose solution was performed at 4 °C for 72 h. Serial frozen sections were made (20 μm thickness) with a freezing microtome (Thermo Fisher Scientific) and prepared for immunostaining. Brain slices were permeabilized with 0.25% Triton-X100 (Beyotime, China) for 15 min, blocked with QuickBlock solution (Beyotime, China) for 15 min at room temperature, and then were incubated with primary antibody at 4 °C overnight. After that, slices were incubated with secondary antibodies for 1 h at room temperature in the dark. A complete list of antibodies used is shown in Additional file 1: Table S2.

For Bodipy staining, after incubating the primary and secondary antibodies, the slices were incubated with Bodipy 493/503 (1:1000 in PBS, ThermoFisher) for 15 min at room temperature. Then slices were sealed with an anti-fluorescence quencher containing 4′,6′-diamidino-2-phenylindole (DAPI). Images were captured using either a fluorescent microscope (Olympus, BX53) or confocal laser scanning microscopy (Olympus, FV1200).

For morphological analysis of microglia, z-stack images (800 × 800 pixels) of brain sections were acquired used on a confocal microscope using a 60 × oil objective. IMARIS 9.0.1 image analysis software (Bitplane, Switzerland) was used for semi-automated image analysis. Based on the previously described [14, 15], morphological analyses of ionized calcium binding adapter molecule 1 (Iba1^+^) microglia were performed. Images were subjected to maximum intensity projection (MIP). 10 to 15 microglia per mouse (*n* = 5/group) were analyzed. Microglial soma area, volume, and sphericity of microglia were determined from surface render images. In addition, microglial solidity and Sholl analysis were performed by Image J. Solidity was calculated by dividing the area of the microglia (Iba1^+^) by their convex area. In microglia with more ramification, the convex area is bigger, which results in a smaller solidity index [16–19].

For quantification of myelin debris engulfment in microglia, images were acquired with an FV1200 laser scanning confocal microscope and analyzed by IMARIS (Bitplane, Switzerland) as previously described [20, 21]. CD68 and Iba1 volumes were quantified by applying 3D surface rendering of confocal z-stacks in their respective channels. To ensure that the data are comparable, the parameters (fix thresholds of intensity and voxel) within each experiment were kept in line and the number of images captured from the lesion in each group was equal. Only dMBP signals, a marker of myelin debris, present within Iba1^+^CD68^+^ structures were counted. Therefore, a new channel for “engulfed dMBP” was created by using the mask function of IMARIS. To eliminate the effects of cell size, the amount of “dMBP engulfed in CD68^+^” was normalized to the total volume of phagocytes in each region (Iba1^+^ total volume). Similarly, for lipid quantification in microglia, only Bodipy signals within Iba1^+^ microglia were counted.

### Histopathological staining

For luxol fast blue (LFB) staining [22], frozen brain sections were dehydrated with gradient ethanol, and immersed in 0.1% LFB (G1030, Servicebio) at 60 °C for 6–8 h. Then, sections were taken out to restore to room temperature, rinsed and differentiated in 0.05% lithium carbonate and 70% alcohol. Sections were then subjected to ethanol gradient dehydration. For Oil Red O (ORO) staining [23], frozen sections were rinsed in 60% isopropyl alcohol for 30 s, stained with freshly prepared ORO solution for 10 min at room temperature, and then washed with 60% isopropyl alcohol to remove excess dye. Rinse the sections well and cover them with glycerin gelatin. Images were taken with a light microscope (Olympus). The area of demyelinating lesions was evaluated by ImageJ (NIH).

### Balance beam test

As previously described [24], the beam apparatus in this study consisted of a beam approximately 0.6 cm or 1.2 cm wide and 100 cm in length suspending 50 cm above some foam pads. A black box was placed at the end of the beam as a guide endpoint. A lamp was placed at the beginning of the beam as a stimulus. Each group of mice was tested in random order. For 2 days before the test, the mice were trained once a day to successfully pass through the entire beam. On the test day, performance on the beam was quantified by measuring the time it took to traverse the beam and the number of paw slips that occur in the process. Before replacing the next mouse, the beam and box were cleaned and wiped down with 75% ethanol wipes.

### Novel object recognition test

In order to minimize the impact of environmental, the day before the test, mice were placed in a rectangular open field of 40 × 40 × 40 cm for 10 min as previously mentioned [25, 26]. On the test day, two identical objects were placed in the back left and right corners of the apparatus, and mice were allowed to adapt to the two objects for 10 min (familiarity stage). One hour later, one of the objects was replaced with another object of a different shape and color. The test lasted 5 min and videos were made to record the time the mice touching the familiar object and novel object. The formula was as follows: The percent of exploring new objects was = TN / (TN + TF), where TN was the time of contacting novel objects and TF was the time of contacting familiar objects. After each experiment, the field and objects were wiped down with 75% ethanol wipes.

### Modified neurological severity score

The neurological function of mice in each group was evaluated by modified neurological severity scores (mNSS), which takes into account motor, sensory, and reflex indicators. The mNSS score is a value ranging from 0 to 18 points. A higher score indicates more severe injury.

### Statistical analysis

Data were displayed as individual dots and mean ± standard deviation (SD). The number of observations (*n*) and the number of biological replicates (mice) (*N*) are provided in the figure legends. Differences between groups were analyzed by Mann–Whitney *U* tests and two-way ANOVA with Bonferroni multiple comparisons by GraphPad Software. *P* value < 0.05 was considered significant.

## Results

### TREM2 is highly expressed in the lesion region of the NMOSD mouse model

**Fig. 1:**
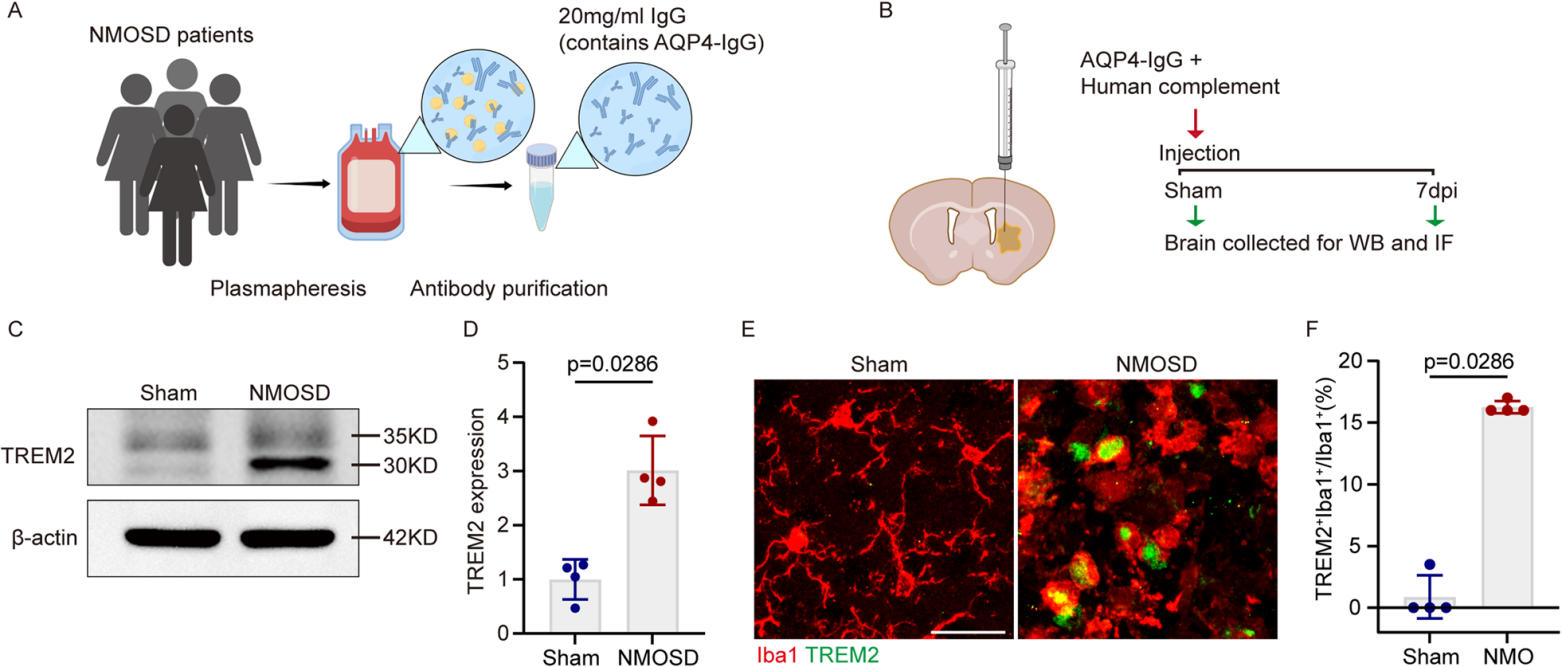
TREM2 is highly expressed in the lesion region of the NMOSD mouse model. **A** Schematic diagram of AQP4-IgG purification (by Figdraw). **B** Schematic of experimental process in mouse model of NMOSD. **C** Representative images of western blotting analysis of TREM2 expression in sham and NMOSD group. **D** Quantification of TREM2 protein relative expression. Data is shown as fold change over the sham group. *N* = 4 mice/group, Mann–Whitney *U* test. **E** Representative images of Iba1 (red) and TREM2 (green) immunostaining in the striatum in sham and NMOSD mice. Scale bar, 30 μm. **F** Quantification of the ratio of TREM2^+^Iba1^+^/ Iba1^+^ cells in sham and NMOSD mice. *N* = 4 mice/group, *n* = 8 fields/group, Mann–Whitney *U* test

To elucidate the role of TREM2 in demyelinating disease, a demyelinating model was established by injecting AQP4-IgG and complement in C57BL/6 mouse brain. Demyelinating damage was induced within 7 days post-injection (dpi) (Fig. 1A, B). We then studied TREM2 expression by Western blotting and immunofluorescence staining. Western blotting showed that TREM2 expression was significantly elevated in the NMOSD group compared to the sham group (Fig. 1C, D). We used the marker Iba1 to display microglia. In parallel, it was found that the TREM2^+^Iba1^+^/ Iba1^+^ ratio was significantly increased in the NMOSD group than that in the sham group by immunofluorescence staining (Fig. 1E, F). These results suggested that TREM2 was highly expressed in microglia after AQP4-IgG and complement injection.

### TREM2 knockout exacerbates CNS damage in the mouse model of NMOSD

**Fig. 2:**
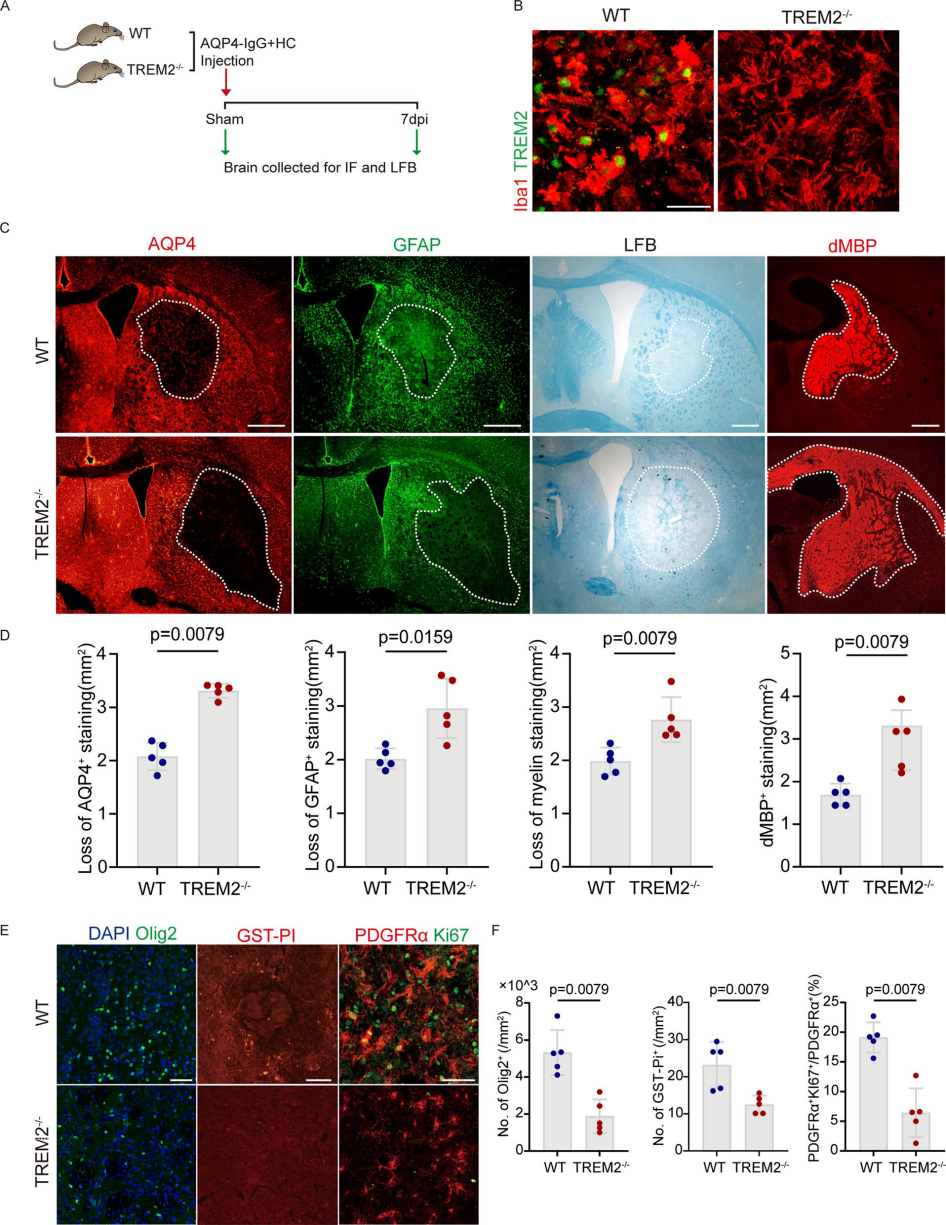
TREM2 knockout exacerbates CNS damage in the mouse model of NMOSD. **A** Timeline of experiment process after AQP4-IgG and human complement injection. Mice were killed after 7 days post-injection (dpi). **B** Representative images of Iba1 (red) and TREM2 (green) immunostaining in the striatum of WT and TREM2^−/−^ mice with NMOSD. Scale bar, 30 μm. **C** Representative images of AQP4 (red), GFAP (green) and dMBP (red) immunostaining and luxol fast blue (LFB) staining in WT and TREM2^−/−^ mice with NMOSD. Scale bar, 500 μm. **D** Quantification of the loss area of AQP4, GFAP and myelin and the area of dMBP staining in WT and TREM2^−/−^ mice with NMOSD. **N** = 5 mice/group, **n** = 10 fields/group, by Mann–Whitney **U** test. **E** Representative images of DAPI (blue), Olig2 (green), PDGFRα (red), Ki67 (green) and GST-PI (red) immunostaining in the striatum of WT and TREM2^−/−^ mice with NMOSD. Scale bar, 50 μm. **F** Quantification of the number of Olig2^+^ cells, the ratio of PDGFRα^+^Ki67^+^/ PDGFRα^+^ cells, and the number of GST-PI^+^ cells in WT and TREM2^−/−^ mice with NMOSD. **N** = 5 mice/group, **n** = 10 fields/group, Mann–Whitney **U** test

To investigate the role of TREM2 in the pathological process NMOSD, we next performed multiple experiments using TREM2^−/−^ and WT mice (Fig. 2A). First, we verified that TREM2 is not expressed in the brain tissue of TREM2^−/−^ mice at 7 dpi by immunofluorescence staining (Fig. 2B). To assess the severity of demyelinating lesions, we analyzed the area of AQP4 and GFAP loss at 7 dpi. Compared with the WT group, the area of AQP4 and GFAP loss was significantly increased in TREM2^−/−^ mice, indicating more extensive astrocyte damage in TREM2^−/−^ mice. LFB staining was used to assess the extent of demyelination, and it was found that the demyelinating area of TREM2^−/−^ mice was significantly increased. Moreover, we measured the area of dMBP accumulation (a marker of degraded myelin basic protein) in TREM2^−/−^ mice, which showed significantly increased myelin debris (Fig. 2C, D).

Olig2 and GST-PI are markers of total oligodendrocyte lineage cells and mature oligodendrocytes (OLs), respectively. PDGFRα is an indicator in OPCs. The number, proliferation, and maturation of oligodendrocytes are key parts of remyelination after injury. We found that the Oligo2^+^ cell count and GST-PI^+^ cell count in the TREM2^−/−^ group were significantly reduced compared to the WT group. In addition, the PDGFRα^+^Ki67^+^/PDGFRα^+^ ratio significantly decreased in the TREM2^−/−^ group (Fig. 2E, F), suggesting that TREM2 deficiency inhibited proliferation of OPCs. Thus, these results indicated that the microglial TREM2 deficiency increased AQP4-IgG and complement-mediated demyelination by hindering myelin repair.

### TREM2 deficiency worsens neurological impairment in the mouse model of NMOSD

**Fig. 3:**
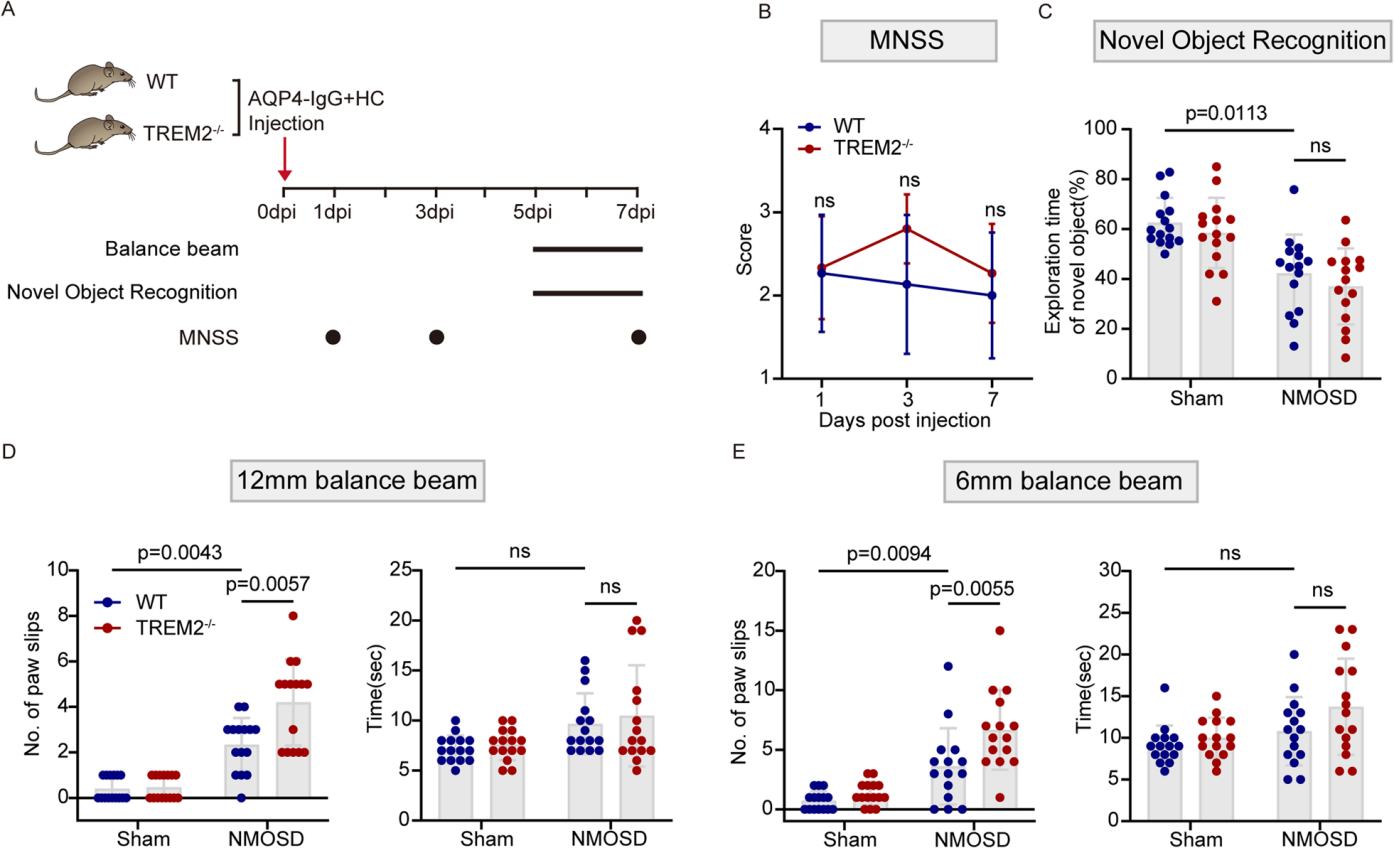
TREM2 deficiency worsens neurological impairment in the mouse model of NMOSD. **A** Timeline of behavior test with or without AQP4-IgG and human complement injection. **B** Quantification of the Modified neurological severity score at 1, 3, 7 dpi of WT and TREM2^−/−^ mice. *N* = 15 mice/group; ns, no significance, two-way ANOVA with Bonferroni multiple comparisons. **C** Quantification of the Novel Object Recognition test of WT and TREM2^−/−^ mice with or without AQP4-IgG and complement injection (7dpi). *N* = 15 mice/group; two-way ANOVA with Bonferroni multiple comparisons. **D** and** E** Quantification of the 12 mm and 6 mm balance beam test of WT and TREM2^−/−^ mice with or without AQP4-IgG and complement injection (7dpi). *N* = 15 mice/group; two-way ANOVA with Bonferroni multiple comparisons

We then scored mNSS on TREM2^−/−^ and WT mice at 1 dpi, 3 dpi, and 7 dpi. In addition, we assessed the learning and memory of mice using the novel object recognition test and motor balance and coordination of mice using the balance beam (Fig. 3A). The results of mNSS showed that TREM2^−/−^ mice scored slightly higher at 3 dpi and 7 dpi, which indicated more severe neurological impairment, but there was no significant difference (Fig. 3B). By the novel object recognition test, we showed the learning and memory ability of the NMOSD mice decreased significantly compared to the sham mice, as reflected by a decrease in the percentage of time spent exploring novel objects, but comparing the TREM2^−/−^ and WT groups at 7dpi, there was no significant difference (Fig. 3C). Moreover, we found that the motor balance and coordination ability of mice were decreased in both the 12 mm and 6 mm balance beams at 7 dpi, as represented by the significantly increased number of paw slips that occurred during the test. The time it took for the mouse to traverse the beam was also increased, but without significant difference (Fig. 3D, E). Comparing TREM2^−/−^ mice with the WT mice, we found that TREM2^−/−^ mice had more paw slips on both thickness sticks than WT mice at 7dpi. Overall, these results suggest that TREM2 deficiency exacerbated motor impairment in mice.

### TREM2 deletion inhibited microglial activation in the mouse model of NMOSD

**Fig. 4:**
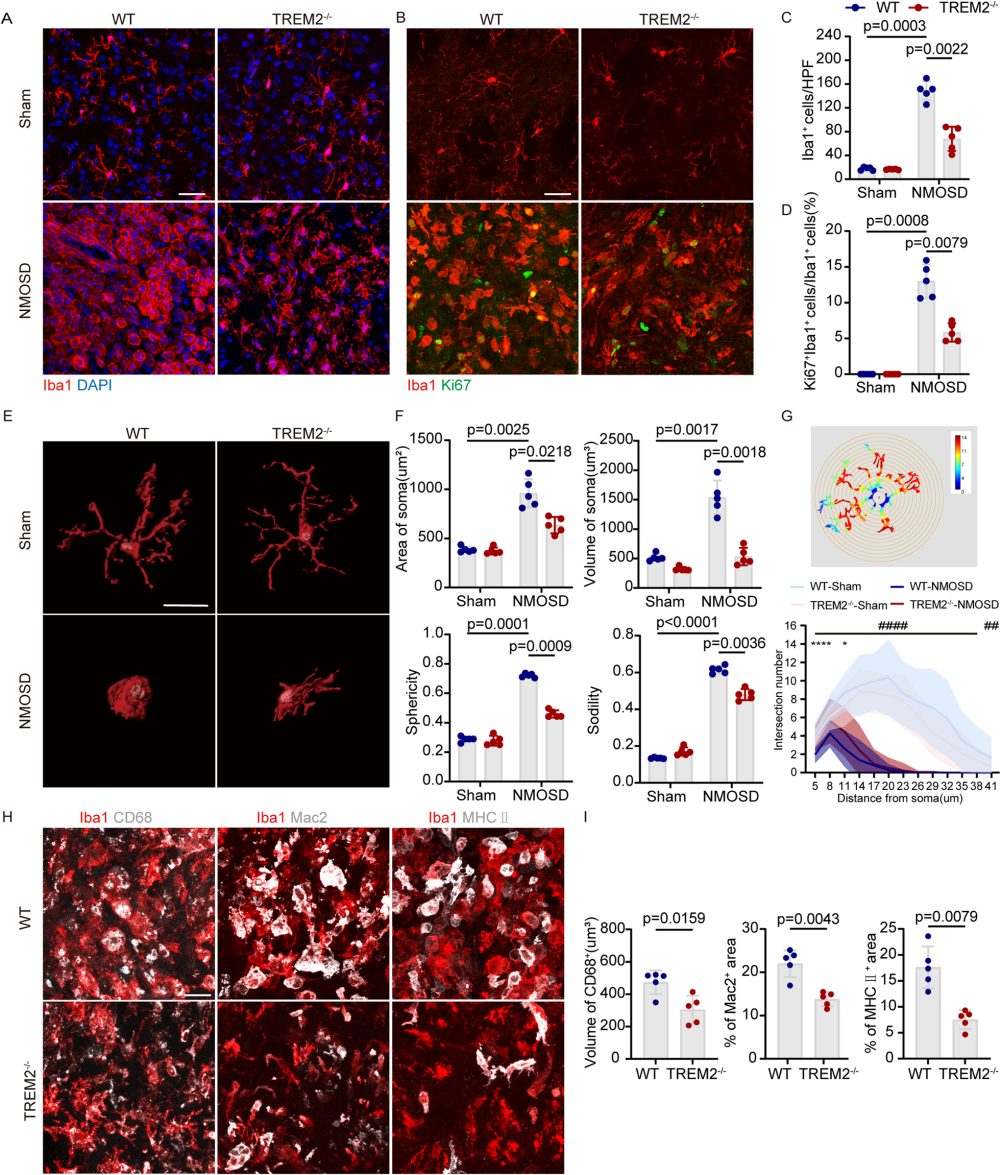
TREM2 deletion inhibited microglia activation in the mouse model of NMOSD. **A** Representative images of Iba1 (red) and DAPI (blue) immunostaining in the striatum of WT and TREM2^−/−^ mice with or without AQP4-IgG and complement injection. Scale bar, 20 μm. **B** Representative images of Iba1 (red) and Ki67 (green) immunostaining in the striatum of WT and TREM2^−/−^ mice with or without AQP4-IgG and complement injection. Scale bar, 20 μm. **C** Quantification of the number of microglia per high-power field (HPF) in the striatum. *N* = 5 mice/group, *n* = 10 fields/group, two-way ANOVA with Bonferroni multiple comparisons. **D** Quantification of the ratio of Ki67^+^Iba1^+^/ Iba1^+^ cells. *N* = 5 mice/group, *n* = 10 fields/group, two-way ANOVA with Bonferroni multiple comparisons. **E** Representative images of the 3D reconstructions of Iba1^+^ cells. Scale bars, 20um. **F** Quantification of the soma volume, soma area, sphericity, and solidity of microglia in the striatum of WT and TREM2^−/−^ mice with or without AQP4-IgG and complement injection. *N* = 5 mice/group, *n* = 10–15 cells/mice, two-way ANOVA with Bonferroni multiple comparisons. **G** Representative images of the intersections masks of Sholl analysis (top) and quantification of the Sholl profiles for four groups (Bottom). Intersections were counted at 4 um intervals from the soma center to a radius of 41 um. *N* = 5 mice/group, *n* = 30 cells/mice, ****p < 0.0001, *p < 0.05, WT-NMOSD mice versus TREM2^−/−^-NMOSD mice; ####*p* < 0.0001, ##*p* < 0.01, WT-Sham versus WT-NMOSD mice, two-way ANOVA with Bonferroni multiple comparisons. **H** Representative images of Iba1, CD68, Mac2 and MHC II stating at 7dpi. Scale bar, 20 μm. **I** Volume of CD68^+^ structures and percentages of Mac2^+^ and MHC II^+^ cells verse total Iba1^+^ cells. *N* = 5 mice for each group, *n* = 10 fields/group, two-way ANOVA with Bonferroni multiple comparisons

TREM2 is expressed specifically by microglia in the CNS, which is believed to be crucial for the clearance of tissue debris in homeostasis and during neurologic diseases [5, 27]. We reasoned that poor pathology and neurologic impairment in the TREM2^−/−^ mouse model of NMOSD might be related to defects in microglial function. The number of microglia was quantified in the striatum of TREM2^−/−^ and WT mice in the sham group and after 7 days of AQP4-IgG and complement injection by immunostaining for Iba1 (Fig. 4A). No significant differences were noted in the number of microglia in the striatum of the sham group. At 7 dpi, accumulation of Iba1^+^ cells was observed in the lesion of WT and TREM2^−/−^ mice compared to the sham group, but significantly decreased in the TREM2^−/−^ mice compared to WT mice (Fig. 4A, C). To further examine microglial proliferation, we performed the co-staining of Ki67 and Iba1. At 7 dpi, the proportion of Iba1 and Ki67 double-positive microglia in the TREM2^−/−^ group was reduced compared to the WT group, suggesting that TREM2 deficiency inhibited microglial proliferation (Fig. 4B, D).

Notably, we also observed differences in microglia morphology in the TREM2^−/−^ and WT groups. It is known that microglia exhibit a ramified morphology in the resting state and an amoeboid morphology in the activated state [28]. Microglia are activated and transform into amoeboid microglia which will have larger cell soma and short, thick branches, corresponding to an increase in the volume, area, sphericity, and solidity of microglia cells [29–31]. We then quantitatively analyzed the soma volume, soma area, and sphericity of microglia by remodeling surfaces to obtain a 3D reconstruction using IMARIS, and analyzed the solidity of microglia by Image J. Our results showed that the soma area, soma volume, sphericity, and solidity of microglia in the lesions of WT and TREM2^−/−^ mice increased at 7dpi compared to sham, while significantly reduced in the TREM2^−/−^ mice compared with WT mice at 7dpi (Fig. 4E, F).

In order to further analyze the effect of TREM2 deficiency on microglial morphology, Sholl analysis was used to evaluate the branching complexity of microglia in the lesion area (Fig. 4G). Microglial branches intersect the circular grid, and fewer intersections indicate a lower complexity of microglial branching, which is a characteristic of microglial activation. We found that the complexity of microglial branching in the lesion area of WT and TREM2^−/−^ mice decreased significantly compared to the sham group at 7dpi. We also found that the branching complexity of microglia in TREM2^−/−^ mice was significantly higher than that in WT mice at 7dpi.

We further analyzed the expression of typical microglial markers, CD68, Mac2, and MHC II, which increases with microglial activation [32–34]. At 7dpi, CD68, Mac2 and MHC II detected in the lesion areas of mice in the TREM2^−/−^ group were significantly lower than those in the WT group (Fig. 4H, I). In summary, TREM2 deficiency inhibited the activation of microglia.

### Phagocytosis of microglia and degradation of myelin debris was attenuated after TREM2 deletion

**Fig. 5:**
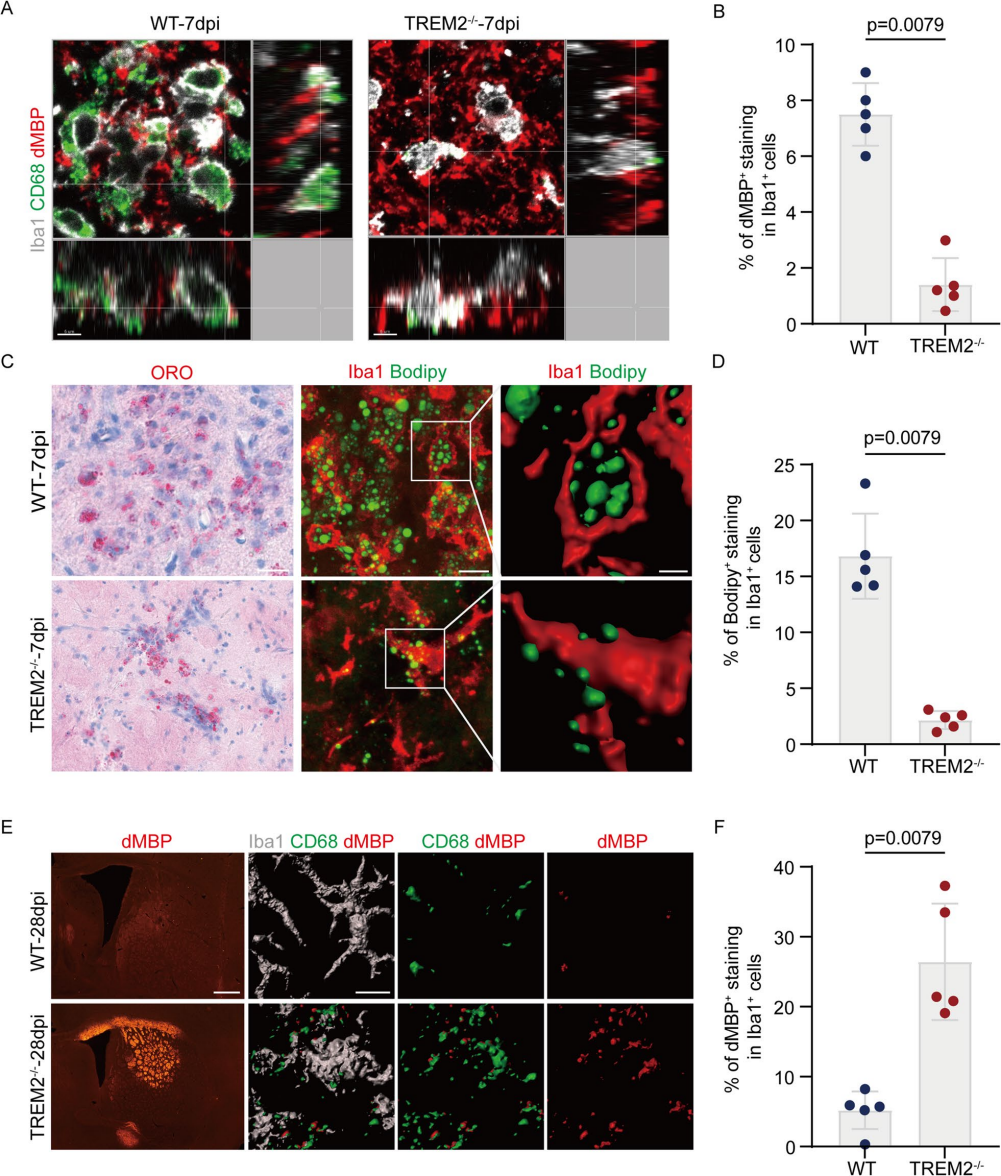
Phagocytosis of microglia and degradation of myelin debris was attenuated after TREM2 deletion. **A** Representative images of Iba1, CD68 and dMBP staining in the striatum of WT and TREM2^−/−^ mice at 7dpi. Scale bar, 5 μm. **B** Quantification of engulfed dMBP within CD68 per microglia in WT and TREM2^−/−^ mice with NMOSD. *N* = 5 mice/group, *n* = 10 fields/group, Mann–Whitney *U* test. **C** Representative images of Oil Red O (ORO) staining (left), Iba1 and Bodipy staining (middle) and the corresponding 3D reconstructions (right) in the striatum of WT and TREM2^−/−^ mice post-NMOSD induction. Scale bars, ORO staining images, 40 μm; fluorescent images, 10 μm; magnified 3D reconstruction images, 3 μm. **D** Quantification of the percentages of the volume of Bodipy^+^ staining within Iba1^+^ cells verse the volume of Iba1^+^ cells. *N* = 5 mice/group, *n* = 10 fields/group, two-way ANOVA with Bonferroni multiple comparisons. **E** Representative images of dMBP immunostaining and 3D surface rendering of confocal images showing volume reconstruction of microglia (gray), CD68 (green) and dMBP (red), as well as dMBP detected within microglial CD68^+^ structures at 28dpi in the striatum of WT and TREM2^−/−^ mice. Scale bar, fluorescent images, 500 μm; confocal images, 10 μm. **F** Quantification of engulfed dMBP within CD68 per microglia at 28dpi in the striatum of WT and TREM2^−/−^ mice. *N* = 5 mice/group, *n* = 10 fields/group, two-way ANOVA with Bonferroni multiple comparisons

Microglia play a key role in myelin debris removal during CNS demyelination [35]. We hypothesized that, in the mouse model of NMOSD, increased lesion areas of dMBP staining in TREM2-deficient mice may be associated with a low number of microglia and a decrease in microglial phagocytosis due to inhibition of activation. To directly assess myelin uptake by microglia, we analyzed the volume proportion of dMBP within the CD68^+^ phagosome structure of Iba1^+^ microglia by immunofluorescence. Quantitative analysis and 3D reconstruction of Iba1^+^ microglia showed that at 7 dpi, the proportion of dMBP volume within the CD68^+^ phagosome structure of TREM2^−/−^ mice was significantly reduced compared with the WT group (Fig. 5A, B). These findings suggested that TREM2 deficiency impaired the phagocytic capacity of microglia.

To further analyze the effect of TREM2 deficiency on the degradation of myelin debris, we used ORO and Bodipy to label neutral lipids which are products of myelin debris degradation [36]. We found that TREM2^−/−^ mice had significantly fewer neutral lipids in Iba1^+^ microglia than in WT mice (Fig. 5C, D). Interestingly, we found that at 28 dpi, dMBP was almost completely cleared in the brains of the WT mice, while a large number of undegraded myelin debris remained in the brains of TREM2^−/−^ mice. We further observed the accumulation of dMBP in microglia at 28dpi and found that dMBP in the phagosome structure of microglia in the WT group was basically absent, while there were still lots of undegraded dMBP in the microglia in the TREM2^−/−^ group, which was significantly larger than that in the WT group (Fig. 5E, F). This provides proof of concept that TREM2 deficiency not only reduces the phagocytosis of myelin debris by microglia, but also reduces the degradation of myelin debris.

## Discussion

In this study, we used AQP4-IgG and human complement to simulate NMOSD pathology and to study the effects of TREM2 in the mouse model of NMOSD. We showed that TREM2 was highly expressed on microglia/macrophages in active demyelinating lesions. We observed that the TREM2 deficiency significantly increased the area of demyelination. Meanwhile, the number of oligodendrocytes and mature oligodendrocytes was reduced, and the proliferation of OPCs was weakened. In addition, our results suggested that TREM2 knockout impaired motor balance and coordination in mice. We then demonstrated that the recruitment and activation of TREM2-deficient microglia were significantly impeded. This resulted in inefficient clearance and degradation of myelin debris, thus demonstrating the critical role of TREM2 in remyelination in NMOSD.

The earliest realization of the important role of TREM2 in human health was after understanding the pathogenesis of Nasu–Hakola disease (NHD), a disease caused by mutations in TREM2 [37]. Subsequent studies have found that TREM2 mutations may increase the susceptibility to the development of various neurodegenerative diseases such as Alzheimer's disease (AD) [38]. This heightened susceptibility is believed to stem from a loss of TREM2 function [39]. On the other hand, studies observed an increase in TREM2 expression in the lesions of neurodegeneration, indicating that TREM2 may play a protective role and contribute to the amelioration of the disease. A study has demonstrated that in patients with multiple sclerosis (MS), TREM2 expression is elevated both at the mRNA and protein levels in active MS lesions [20]. And it has been suggested that TREM2 is mainly expressed in myelin-loaded macrophages (called foamy macrophages) [40]. Similarly, in our study, we showed that high TREM2 expression occurs in microglia in NMOSD mouse lesions. In addition, our findings indicate that in NMOSD, the activation of microglia is accompanied by a rise in TREM2 expression, leading to a significant improvement in the phagocytic ability of the microglia.

Growing evidence suggests that TREM2 plays a beneficial role in neurodegenerative diseases. It was reported that in cuprizone (CPZ) model of CNS demyelination [20], THY-Tau22 transgenic line [41], and experimental autoimmune encephalomyelitis (EAE) [40, 42], TREM2 was protective. Our results also support this conclusion. In the NMOSD mouse model, TREM2 deficiency leads to more severe demyelination and motor impairment. We hypothesized that this might be caused by a hindrance in remyelination, as a decrease in the number of OPCs and OLs and a reduction in the proliferation of OPCs were observed. Effective removal of myelin debris is essential to eliminate inhibitors that interfere with OPCs activation, recruitment to demyelination sites, and differentiation into myelin maturation OLs [43], which is critical for remyelination.

The general consensus is that TREM2 is essential for inducing transcriptomic and functional processes in disease-associated microglia (DAMs), a subtype of microglia that occur in the brain in various animal models of neurodegeneration, characterized by the activation of pathways related to phagocytosis and lipid metabolism [44, 45]. Consistent with previous studies [46], we found that TREM2 deficiency reduced the density and proliferation of microglia in the lesions. In addition, we further analyzed the activation status of microglia in the demyelinating lesions. Our results showed that the lack of TREM2 in mice resulted in the defective activation of microglia, which affected the phagocytosis and degradation of myelin debris by microglia. Furthermore, at 28 dpi, a time point when most myelin debris have been degraded in microglia in WT group, we found that in TREM2-deficient mice, dMBP remained abundant, and the microglia did not return to a resting state. The persistent presence of phagosomes and dMBP in TREM2^−/−^ microglia at 28 dpi indicates that the lack of TREM2 impairs the ability of microglia to degrade myelin debris. It is possible that the lack of TREM2 leads to lipid metabolism defects, resulting in the long-term accumulation of myelin debris in microglia that cannot be effectively cleared. This could make it extremely challenging for remyelination. Cantoni et al. have reported that TREM2 deficiency leads to lipid metabolism defects in the CPZ model, and Garyfallia et al. have demonstrated that TREM2 deficiency impairs lipid droplet biogenesis, providing further evidence to support our findings [46, 47]. Elucidating the role of TREM2 may open up new avenues for therapeutic intervention in demyelinating diseases of the central nervous system.

## Conclusion

**Fig. 6:**
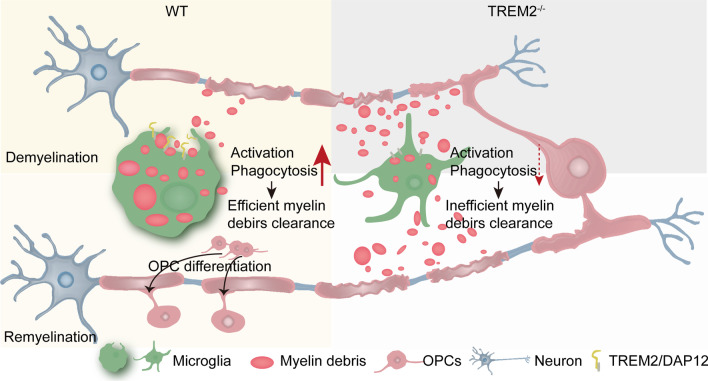
Graphic abstract. The absence of TREM2 results in diminished activation of microglia, which obstructs the phagocytic process and degradation of myelin debris by these cells. As a result, the repair of the myelin sheath by oligodendrocytes is impeded

In summary, our findings support that TREM2 is important for microglial activation and promotes microglial phagocytosis and degradation of myelin debris, playing a neuroprotective role during the demyelination of NMOSD (Fig. 6).

## Supplementary Information

**Additional file 1: Table S1.** Clinical characteristics of NMOSD patients for purified AQP4-IgG. **Table S2.** A list of materials. **Fig S1.** Fullblot images.

## Data Availability

The original contributions presented in the study are included in the article, further inquiries can be directed to the corresponding authors.

## Abbreviations

NMOSD: Neuromyelitis optica spectrum disorder
CNS: Central nervous system
TREM2: Triggering receptor expressed on myeloid cells 2
AQP4: Aquaporin-4
OPCs: Oligodendrocyte precursor cells
PBS: Phosphate-buffered saline
WT: Wild-type
NC: Nitrocellulose
DAPI: 4′,6′-Diamidino-2-phenylindole
Iba1: Ionized calcium binding adapter molecule 1
MIP: Maximum intensity projection
LFB: For luxol fast blue
ORO: Oil Red O
mNSS: Modified neurological severity scores
SD: Standard deviation
dpi: Days post-injection
OLs: Oligodendrocytes
NHD: Nasu–Hakola disease
AD: Alzheimer's disease
MS: Multiple sclerosis
CPZ: Cuprizone
EAE: Encephalomyelitis
DAMs: Disease-associated microglia
