# Supplementary material for: TREM2 deficiency inhibits microglial activation and aggravates demyelinating injury in neuromyelitis optica spectrum disorder
Source: J Neuroinflammation. 2023 Apr 3;20:89. doi: 10.1186/s12974-023-02772-3 (PMC10069075; doi:10.1186/s12974-023-02772-3)

Table S1. Clinical characteristics of NMOSD patients for purified AQP4-IgG.

Patient No.	Gender	Serum anti-AQP4 antibody (CBA)
NMOSD-1	Female	1:3200
NMOSD-2	Female	1:1000
NMOSD-3	Female	1:320
NMOSD-4	Female	1:320

CBA, cell-based assay.

Table S2. A list of materials.

Category	Source	Identifier
**Antibodies**		
Iba-1	WAKO 1:500 (IF)	Cat#019-19741
Iba-1	Abcam 1:500 (IF)	Cat#ab5076
TREM2	R&D system 1:1000 (WB) 1:100 (IF)	Cat#AF1729
beta-Actin	Proteintech 1:4000 (WB)	Cat#66009-1-Ig
AQP4	Proteintech 1:100 (IF)	Cat#16473-1-AP
GFAP	CST 1:400 (IF)	Cat#3670
dMBP	Millipore 1:1000 (IF)	Cat#AB5864
Olig2	Proteintech 1:200 (IF)	Cat#13999-1-AP
GST-pi	MBL 1:200 (IF)	Cat#311
PDGFRα	CST 1:500 (IF)	Cat#3174
Ki67	Invitrogen 1:500 (IF)	Cat#14-5698-37
CD68	Bio-Rad 1:500 (IF)	Cat#1957
Mac2	Biolegend 1:200 (IF)	Cat#125401
MHC II	Invitrogen 1:200 (IF)	Cat#MA5-41026
Cy3-AffiniPure goat Anti-Rabbit	Jackson Immuno Research	Cat#111-165-003
Donkey anti-sheep Alexa Fluor 488	Jackson Immuno Research	Cat#713-545-147
Donkey anti-mouse Alexa Fluor 488	Jackson Immuno Research	Cat#715-545-150
Donkey anti-mouse Alexa Fluor 594	Jackson Immuno Research	Cat#715-585-150
Donkey anti-rabbit Alexa Fluor 488	Jackson Immuno Research	Cat#711-545-152
Donkey anti-rat Alexa Fluor 488	Jackson Immuno Research	Cat#712-545-150
Donkey anti-rat Alexa Fluor 594	Jackson Immuno Research	Cat#712-585-150
Donkey anti-rat Alexa Fluor 647	Jackson Immuno Research	Cat#712-605-150
Donkey anti-goat Alexa Fluor 488	Jackson Immuno Research	Cat#705-545-147
HRP Goat Anti-Rabbit IgG (H+L)	Abclonal	Cat#AS014
HRP Goat Anti-Mouse IgG (H+L)	Abclonal	Cat#AS003
**Reagents**		
RIPA Lysis buffer	Boster	Cat#AR0102

PMSF	Boster	Cat#AR1178
Cocktail	MCE	Cat#HY-K0010
Protein marker (10-180kDa)	Thermofisher	Cat#26616
5X Loading buffer	Solarbio	Cat#P1040
BCA protein assay kit	Boster	Cat#AR1189
ECL Enhanced Kit	Abclonal	Cat#RM00021
BODIPY™ FL	ThermoFisher	Cat#D2184
Neutral red	Sigma	Cat#N4638
LFB staining kit	ServiceBio	Cat#G1030
Oil red O staining	Baso	Cat#BA4081
Neutral resins	Baso	Cat#BA7004
Pooled Human Complement Serum	Innovative research	Cat#ICSER10ML
Isoflurane	RWD Life Science	Cat#R510-22-10
OCT compound	SAKURA	Cat#4583
**Animals**		
WT C57BL/6 mice	GemPharmatech (Nanjing, China).	Strain NO.N000295
TREM2$^{-/-}$ mice	This paper	Gifted

Fig S1. Fullblot images.

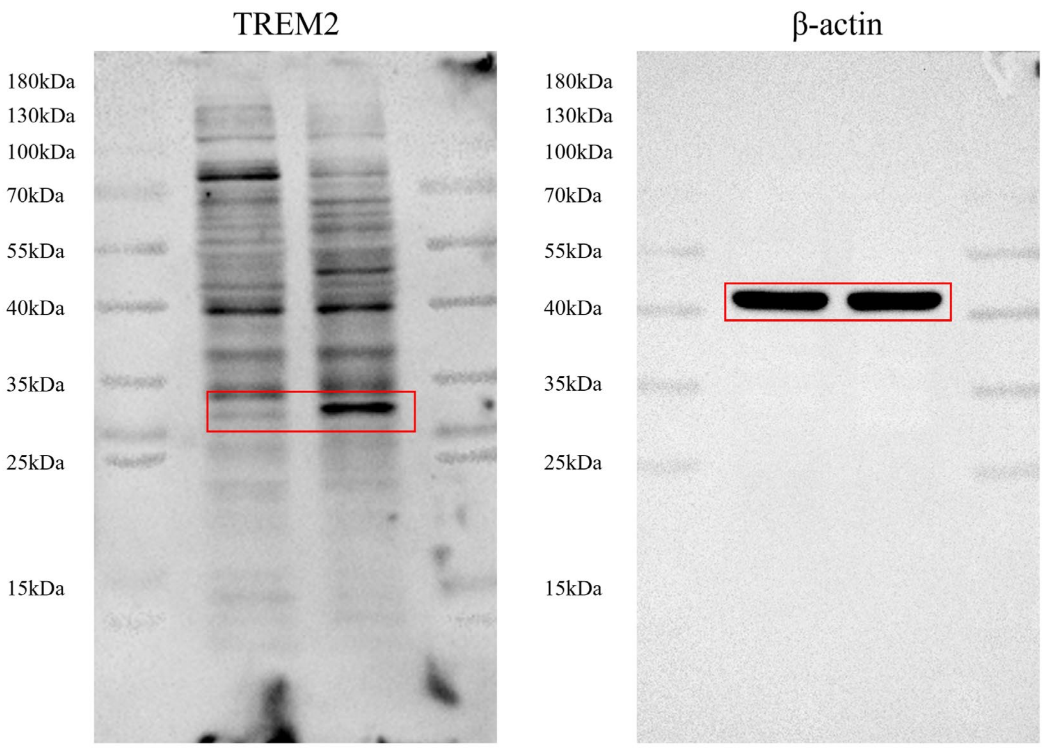

Supplement: Supplementary file 1 — Additional file 1: Table S1. Clinical characteristics of NMOSD patients for purified AQP4-IgG. Table S2. A list of materials. Fig S1. Fullblot images. [file 12974_2023_2772_MOESM1_ESM.pdf]
